# The Perspective of Rural Physicians Providing Abortion in Canada: Qualitative Findings of the BC Abortion Providers Survey (BCAPS)

**DOI:** 10.1371/journal.pone.0067070

**Published:** 2013-06-28

**Authors:** Jennifer Dressler, Nanamma Maughn, Judith A. Soon, Wendy V. Norman

**Affiliations:** 1 Women’s Health Research Institute, British Columbia Women’s Hospital, Provincial Health Services Authority, Vancouver, Canada; 2 Faculty of Pharmaceutical Sciences, University of British Columbia, Vancouver, Canada; 3 Department of Family Practice, University of British Columbia, Vancouver, Canada; Indiana University, United States of America

## Abstract

**Background:**

An increasing proportion of Canadian induced abortions are performed in large urban areas. For unknown reasons the number of rural abortion providers in Canadian provinces, such as British Columbia (BC), has declined substantially. This study explored the experiences of BC rural and urban physicians providing abortion services.

**Methods:**

The mixed methods BC Abortion Providers Survey employed self-administered questionnaires, distributed to all known current and some past BC abortion providers in 2011. The optional semi-structured interviews are the focus of this analysis. Interview questions probed the experiences, facilitators and challenges faced by abortion providers, and their future intentions. Interviews were transcribed and analyzed using cross-case and thematic analysis.

**Results:**

Twenty interviews were completed and transcribed, representing 13/27 (48.1%) rural abortion providers, and 7/19 (36.8%) of urban providers in BC. Emerging themes differed between urban and rural providers. Most urban providers worked within clinics and reported a supportive environment. Rural physicians, all providing surgical abortions within hospitals, reported challenging barriers to provision including operating room scheduling, anesthetist and nursing logistical issues, high demand for services, professional isolation, and scarcity of replacement abortion providers. Many rural providers identified a need to “fly under the radar” in their small community.

**Discussion:**

This first study of experiences among rural and urban abortion providers in Canada identifies addressable challenges faced by rural physicians. Rural providers expressed a need for increased support from hospital administration and policy. Further challenges identified include a desire for continuing professional education opportunities, and for available replacement providers.

## Introduction

Induced abortion is a common procedure in Canada [1]. Currently 31% of Canadian women have had at least one abortion [2]. Induced abortion or simply “abortion” is defined by the Canadian Institute for Health Information as “the medical termination of pregnancy” [1]. There are many factors that affect access to abortion in Canada, where only physicians are licensed to perform abortions. From a geographical perspective, access to abortion in Canada can be challenging, with potentially large distances between women and where abortion services are offered [3], [4].

Traditionally, abortions were performed only in hospitals across Canada. Since the Supreme Court of Canada *R. vs. Morgentaler* decision in 1988, [5] abortion service has been available in non-hospital clinics in many provinces, however these are exclusively located in large urban areas [4]. In BC it is estimated that the proportion of abortions performed in rural or community hospitals decreased by at least 60% between 1995 and 2005 [6]. Increased distance to abortion providers is a major barrier to women accessing abortions [3], [7], [8].

The attrition of abortion providers in North America is a well-described phenomenon [9], [10]. In the US, the number of clinicians available to provide abortions declined by 38% between 1982 and 2000 [7], [11]. In BC, the Pregnancy Options Service (POS) provides province-wide referral to the closest abortion provider through a toll-free telephone line. The POS database indicates a 62% decline in the number of registered individual rural abortion providers between 1998 and 2005 [6]. The retirement of providers without replacement, (i.e. ‘graying’) first described 20 years ago, is a factor in the decline of rural abortion providers in the US [8]. The reasons for the decline in Canada are currently unknown.

Currently, no studies have investigated the experiences of individual Canadian abortion providers. The goal of this study was to gain insight into the challenges and barriers faced by current BC providers. The objectives were threefold: 1. To identify and explore the major barriers to abortion service delivery from the perspectives of abortion providers. 2. To determine if these perspectives differed between urban and rural providers. 3. To explore the future intentions of providers, including factors affecting long-term availability and the potential for training replacement providers.

This study is the first in Canada to examine the challenges and experiences of both rural and urban abortion providers, and will provide information to inform planning for continuing professional education and health service provision.

## Methods

### Ethics

Ethics board approval was obtained from the University of British Columbia Children’s and Women’s Hospital Research Ethics Board (H11-00766) prior to commencement of the study. All participants submitted written completed surveys, including a statement that submission of the survey would imply consent to participation.

### Participants and Setting

This study is part of a mixed methods study examining clinical practices, attitudes and experiences of BC abortion providers (BCAPS). The study included completion of self-administered written questionnaires and voluntary participation in subsequent semi-structured interviews following a defined script (See Supplemental Material S1: BCAPS Interview Script). The qualitative findings from the interview portion of BCAPS are reported here. Please see the companion article in this issue, “Barriers to Rural Induced Abortion services in Canada: Findings of the British Columbia Abortion Providers Survey (BCAPS)”, for the quantitative survey results.

Questionnaires were distributed to all 46 current BC surgical abortion providers, and four past providers, listed on the POS registry. Questionnaire participants were invited to partake in a brief, semi-structured, audio recorded face-to-face or telephone interview at their convenience. Survey respondents included 39/46 (85%) of current providers and two of the four former abortion providers sampled. Twenty-nine (70.7%) of these physicians volunteered for semi-structured interviews. Volunteers were contacted, using the preferred contact information they provided, to arrange a convenient time for a telephone or in-person interview. Non-responding volunteers were contacted up to four times.

### Design

Co-investigators (JD and NM) and two research assistants conducted semi-structured interviews with respondents. Interviews gathered basic demographic information through a series of short, closed-ended questions, then focused on the challenges participants face as abortion providers in their communities, specifically administrative and personal barriers, including harassment. Additional questions addressed participants’ intentions for future provision of abortion services.

Interviews were recorded and then transcribed by a confidential transcription service which removed identifying information. Participants were identified by either urban or rural codes. “Urban” participants were defined as those in a Census Metropolitan Area (CMA). Statistics Canada defines a CMA as being “formed by one or more adjacent municipalities centred on a large urban area (known as the urban core). The census population count of the urban core is at least 100,000 to form a census metropolitan area. To be included in the CMA, other adjacent municipalities must have a high degree of integration with the central urban area, as measured by commuting flows derived from census place of work data.” [12] All other providers were considered rural. All interviews were completed between April 28, 2011 and February 14, 2012.

A thematic analysis was used to examine and analyze the interview transcripts. Transcripts were independently coded into analytical themes by co-investigators (JD and NM) and results were compared. Themes coded discrepantly were reviewed by both investigators until a common theme was agreed upon. The material was then reviewed by all authors for thematic saturation. Results from rural and urban groups were then compared.

## Findings

### Participant Characteristics

Among the 29 volunteers, 23 interviews were completed, and of these 20 recordings survived to transcription, including 13 rural and seven urban participants. One digital recorder, containing four interviews, was subjected to leaking battery acid, causing a loss of the four recordings. Only one of these interviews was able to be re-done. Final analysis thus included interviews from 48.1% of rural abortion providers, and 36.8% of urban providers on the BC POS database. Urban providers interviewed were more likely to be providing abortions for a longer period of time, with a minimum of 15 years. While several of the rural providers interviewed had been performing abortions for more than 20 years, 8/13 (61.5%) had been providing abortions for six years or less.

Three main themes were identified within the rural participant narratives: 1. Hospital and logistical barriers to provision, 2. Professional isolation and need for discretion within a rural community, and 3. Lack of replacement providers, including barriers to training opportunities. Urban providers, in general, reported few barriers to provision and ample availability of training opportunities and replacement providers.

### Hospital and Logistical Challenges

There were striking differences between the urban and rural physicians when discussing the challenges faced as an abortion provider. In general, the urban abortion providers faced fewer or no barriers to provision. According to one urban physician, “I felt, and have always felt, very supported within my office and within the urban abortion facilities where I work. My colleagues and the administrators at [the] hospital and facilities are very ‘on side’.” The challenges that did emerge for some of the urban providers included lack of operating room time for those providing hospital-based services, and increasing restrictions on the basic funding support for the urban purpose-specific clinics (“abortion clinics”). As one urban physician stated:

“…not so much that the funding is going to be threatened to the service as a whole but it may be threatened to the organization where I work.”

Conversely, rural participants faced many challenges to provide abortion service in their communities. Unlike their urban counterparts who work in purpose-specific abortion clinics, all rural participants performed abortions within the local hospital operating room. The barriers associated with this setting included lack of operating room time for abortions, a tendency to defer an abortion case for an “urgent” non-abortion case, and difficulties in logistically scheduling operating room staff (e.g., nurses and anesthesiologists) to accommodate staff who did not wish to participate in abortion care. Several rural physicians faced logistical challenges when scheduling patients for counseling (occurring at their private practice offices), timely ultrasounds and for procedures. Two participants described difficulty starting to provide abortion service in a small community. For example, one rural provider stated:

“The anesthetists…just don’t want to be in my room because they don’t get enough money per case and sometimes my case doesn’t run. I’m expendable. If there’s no anesthetists… well, ‘It’s okay we’ll just pull the anesthetist out of [participant]’s room’. So there’s a bit of a judgment call there on anesthesia…there are people that have ideas about what they think is more important.”

Typically, rural abortion providers are required to fit into their private practice office time the counseling and pre-operative assessment that would be performed by allied health professionals in the interdisciplinary urban abortion clinics. For example, one participant stated, “You know, at a freestanding [urban] abortion clinic, they have counselors that do a lot of the counseling with the patients. So actually you [one physician] can provide a lot more care to a larger group of women.”

### Isolation

Among rural participants, a common concern identified was professional and personal isolation as an abortion provider. Most rural physicians interviewed were the sole provider for abortion service in their communities, some serving a large catchment area. Several physicians indicated feeling overwhelmed by their inability to meet local requirements for abortion service in a timely manner due to facility restrictions. Some noted waiting lists in excess of five weeks from first contact until the procedure could be performed. Two physicians described pressure to always be available as the sole abortion provider in their community. One participant discontinued his/her surgical abortion practice because s/he was unable to find another physician to assist in providing 24 hour availability for emergency care in case of a complication. Another participant discussed his/her frustration with the isolation as follows:

“Biggest barriers I see, and things that might see me stopping, is the sheer volume. And if it’s only me trying to see everyone, with no breaks and, you know, to feel like you can’t even take a week away because, either it’d pile up or people aren’t going to be able to be seen…The biggest barrier is just…keeping myself from getting burnt out, providing the services and feeling like I can’t do as much as I want to.”

In addition to facing high demand, many rural physicians described personal isolation as a need to “fly under the radar” or “keep a low profile”. One abortion provider described the fear of the impact on his/her children if community members knew about his/her practice. S/he concluded, “And so, I think we do try to fly below the radar a bit in a smaller community.” Another physician avoided going to the local [place of worship] due to the size of the community and the fear of being judged. S/he stated:

“…I think the only thing is, I don’t go to church, and I don’t practice religion, just because it’s a small community, and myself, I wouldn’t want to be seen as – or even I don’t want to perceive myself as – somebody with …a double standard where I believe in a religion and all of its tenets and then I perform abortions. I think I’d rather just keep to myself and so I do avoid church because of how small the community is.”

Another rural physician described his/her feeling of isolation:

“I feel like I’m justifying what I’m doing … it’s so hard not to get caught up with all the negative publicity and the politics that somehow what you’re doing is shameful or bad, when it’s not, it’s just healthcare. So, you know, having someone else in the community would be nice.”

Although this was a theme that was more common among rural providers, one urban provider stated “I do feel a very urgent and continual need to be quite secretive about my work in general.” Most urban providers described a sense of security in their relative anonymity. For example, one urban physician stated:

“I think I’m in a really privileged position being a provider in a large urban center where I can kind of hide and not many people need to know what I do.”

Additionally, physicians providing abortion service in rural communities lack professional support in the form of easily accessible continuing professional education events and camaraderie with other professionals providing abortion service. Rural abortion providers have limited opportunities to attend topic specific continuing professional education events among other abortion provider participants. One participant highlighted the importance of these events in the following statement:

“Sometimes I feel like I’m operating or I’m working in isolation. And so coming to [interview took place at a provincial abortion meeting] these kinds of meetings, I go to the [name of abortion providers’ organization] conference every other year…It’s nice to be in a situation where you’re with a lot of like-minded people and you can just sort of talk freely about issues.”

Another rural physician discontinued his/her surgical practice, in part because s/he felt s/he was not providing women with an equivalent service to an urban clinic, and was unable to obtain updated training to reinforce his/her skills. S/he explained: “I don’t feel like I’m providing a good service, an equivalent service to what women would get if they were in [clinic name] or at a women’s clinic down in [the major urban center]…”.

### Training and Replacement

Many urban abortion providers described no concerns with the availability of other physicians to replace their services. One participant stated, “I think in [urban facility], [my services] could easily be replaced, there are many physicians who would like to work at [urban facility] but there just is not the space at the time. So, in the [city name] area I don’t think it would be much of an issue.” This perceived availability of replacements was less pronounced in the smaller urban centers and among the urban providers who performed second trimester abortions.

With respect to training new physician replacements, many urban providers described an established training program through the local university-based medical school, or having participated in the provision of abortion training for family practice or obstetrician-gynecology residents and rural physicians. One urban provider expressed frustration that he/she had trained many physicians who did not go on to provide abortion services, and one provider stated that he/she trained many who could not attain a position to provide abortion at one of the urban abortion clinics in the city in which they trained.

Rural physicians perceived a lack of available replacements. One physician stated, “Nobody would ever [provide abortion] here. I’m the only one. We approached other people, like the other physicians, and there’s nobody interested in doing it.” Rural physicians were less likely to train other physicians in skills for provision of abortion, in their communities. One participant described a feeling of insecurity in training another physician, particularly in light of the lack of specialist back up in the event of a complication. As well, two physicians described a lack of volume of abortion cases as a deterrent to the local training of new abortion providers. “I was hoping to get this [physician] trained but I think [the physician] is going to have to go to a[n urban] clinic where there are several cases a day, so [the physician] can get [many] cases in … if it’s going to have any chance of being successful.”.

## Discussion

While several studies have demonstrated the decline in abortion providers in North America,[6], [7], [9]–[11] this is the first study to explore the experiences of rural and urban providers in Canada. Additionally, it is the first to identify the challenges to provision and the specific barriers faced by physicians providing abortions within rural compared to urban communities.

While abortions in urban centers are increasingly performed in purpose-specific high volume clinics, abortions in BC rural communities continue to take place predominantly within the hospital operating room. This limitation creates daunting challenges for rural abortion provider physicians including logistical limitations on operating room time, and availability of anesthesia and nursing staff. Alternatively, urban abortion provider physicians, most notably those practicing in urban purpose-specific clinics, identified few logistical challenges.

Rural participants expressed feeling increased isolation compared to their urban counterparts. This manifests as additional responsibility for abortion provider physicians within a rural community to be available at all times and is inherently accompanied by a lack of professional support. Rural abortion providers expressed a need for discretion within their community, further contributing to professional and personal isolation. Thus, rural physician participants identified their need for additional support to facilitate continuing professional education and networking with other abortion providers.

Training of new abortion providers from among physicians already in practice, or planning to practice, in rural communities could help to decrease the sense of professional isolation faced by physicians providing abortion service in rural areas. Rural providers may be willing, but low volumes and logistical considerations in operating rooms limit their ability to train new providers within their own communities. Expansion of current urban abortion service training programs for physicians, and active recruitment and incentives for existing and new physicians to provide abortion, are needed. While family practice and gynecology physicians are more likely to provide abortion service if exposed to abortion training in residency, [13]–[23] those physicians currently practicing in rural areas may have limited exposure to abortion training. This may limit rural physicians’ willingness to provide abortion. Support for initial training, and particularly for continued professional education for practicing rural abortion providers, could alleviate many of the concerns expressed.

Future research could compare challenges identified in this study with those among urban and rural abortion providers in other regions of Canada, and in other countries.

Limitations in this study include factors leading to potential for sampling bias. The study may have been limited by sampling of those physicians registered with the BC POS. Although the POS is thought to maintain a comprehensive list of physicians in the province providing surgical abortions, POS recognizes there is neither the need, nor is it feasible, to maintain a list of all physicians who provide a medication-induced abortion (“medical abortion”). For example, we interviewed many surgical abortion providers who also provide medical abortions, but we did not seek out physicians who may be providing exclusively medical abortions.

This is the first Canadian research to examine factors influencing rural and urban physicians providing abortion services. The study identifies additional challenges faced by rural BC abortion providers compared to their urban counterparts. Key health system factors potentially contributing to the declining availability of rural abortion services include a perceived lack of administrative, hospital and collegial support, and limited support for continued professional education and trained replacement providers.

## Supporting Information

Supplemental Material S1 **BCAPS Interview Script.**
(DOC)
