# Supplementary material for: The Perspective of Rural Physicians Providing Abortion in Canada: Qualitative Findings of the BC Abortion Providers Survey (BCAPS)
Source: PLoS One. 2013 Jun 28;8(6):e67070. doi: 10.1371/journal.pone.0067070 (PMC3695949; doi:10.1371/journal.pone.0067070)
Supplement: Supplemental Material S1 — BCAPS Interview Script. (DOC) [file pone.0067070.s001.doc]

**
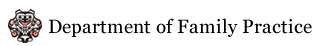
**

**BC Abortion Providers Survey**

**Script for abortion provider interviews**

Introduction

Thank you for completing our survey about Abortion provision in BC and for agreeing to participate in this brief in person interview. My name is __________. I am a:

- research assistant in the Department of Family Practice at UBC *or*
- resident in the family medicine program in Kelowna

and will be conducting your interview today. I expect that the interview will take approximately 10 to 15 minutes to complete.

For this interview, we have prepared a series of questions, designed to build on the paper survey which you completed. The purpose of the interview is to explore personal experiences and intentions of abortion providers in BC.

I will be recording the interview. Your responses will be recorded digitally and transcribed by a confidential service, removing personal information such as your name and the name of the town or city in which you practice, before analysis to ensure confidentiality. All digital audio files will be deleted after transcription.

Your participation in this interview is entirely voluntary and you may refuse to participate or discontinue the interview at any time without penalty. By completing the interview, you have consented to our use of the information you provide.

1. How many years have you been in practice since residency?

2. How many years have you been providing abortion?

*and for those who were recently abortion providers but have stopped:* When did you stop providing abortions?

3. Approximately how many abortions do/did you provide per month?

4. Now I would like to ask you if you feel there are any general or specific challenges you face(d) as an abortion provider?

Prompt:

1. What challenges or difficulties have you faced professionally either in your relationship with administrators or at your hospital or clinic, or in your own office with management of your office staff?
2. Have you or your family or colleagues had to face challenges that you would describe as harassment or interpersonal conflict with the community, your colleagues or with other health care personnel?
3. Have you encountered, or do you anticipate any other factors that you feel could discourage you from providing abortions?

5. Have you ever considered discontinuing your practice as a provider?

If no: What factors may cause you to discontinue your practice as a provider?

If yes: Which factors have led you to consider making this decision?

6. If something were to happen to prevent you from providing abortions in this community, can you speculate on whether, how, or if your service could be replaced? (*for those who are no longer providing we would ask*: Do you know or can you speculate on what options your community will have to replace this service?)

If elaboration is needed, prompt for:

1. Availability of other providers nearby
2. Plans for replacement/training of another physician willing to provide

Thanks very much for taking the time to talk with me and for your contribution to our study. If you have any questions, please feel free to contact Dr Wendy Norman, whose contact information is provided in your survey package.
